# Re: Estimating and reducing dose received by cardiac devices for patients undergoing radiotherapy

**DOI:** 10.1120/jacmp.v17i4.6321

**Published:** 2016-07-08

**Authors:** Dimitris N. Mihailidis

**Affiliations:** ^1^ Medical Physics Solutions, LLC and West Virginia University‐School of Medicine Charleston Division Charleston WV USA

To the Editor:

I read with great interest the manuscript of Bourgouin et al.,^(1)^ describing methods to estimate and/or measure out‐of‐field doses to a cardiac implantable electronic device (CIED) for patients that undergo radiotherapy. This is a topic that deserves great attention in today's radiotherapy practice since a great number of cancer patients who will receive radiation have a pacemaker or implantable cardioverter‐defibrillator. For this reason, publications like the one under discussion,^(1)^ might be used as guidance to practicing professionals. The purpose of that manuscript was the evaluation of the dosimetric effect that a sheet of lead shield over a CIED has, based on out‐of‐field dose measurements at the location of the CIED, as a way to reduce the dose to the CIED.

There are several discrepancies and inaccuracies in the manuscript that I would like to point out. Equation (1) in the manuscript is inaccurate and is missing the radial distance dependence, r. Parameter a of Eq. (1) mostly depends on the irradiated area size, but not proportionally and, on the primary photon energy, then, parameter b is practically constant with photon energy representing the attenuation of scattered photons in water.^(2)^ I believe Eq. (1) in the article should read

(1) $$D=ae^{-br}+c.$$

The authors claim that the Exradin W1 scintillator detector was calibrated according to manufacturer's method. The setup for out‐of‐field dose measurements demands careful calibration of the detectors used in an out‐of‐field geometry. For out‐of‐field dosimetry, lower energy scattered photons are more significant for the out‐of‐field spectrum, which typically peaks at a few hundred keV. That spectrum potentially creates energy dependencies in the detectors used, resulting erroneous dose estimates for out‐of‐field doses if the in‐field calibration is used.^(3)^In addition, out‐of‐field measured doses are typically very low doses as one moves away from the treatment area (the in‐phantom scatter component decreases and leakage dominates). The authors' data presentation and conclusions have not taken into account the above effects, resulting in several inaccuracies. For example, out‐of‐field doses for 6 MV primary photon beam compared to 23 MV are not noticeably higher, especially for small lateral distances that concern a CIED. The data presented in their manuscript are in contradiction with AAPM Task Group No. 36^(4)^ and more recent data by Kry^(5)^ and Crofor et al.,^(6)^ which show a very weak primary energy dependence (some data show higher out‐of‐field doses for 6 MV compared to 18 MV photon beams). One important point for CIED dose evaluation is that, for photon energies higher than 10 MV, the out‐of‐field secondary neutron doses are more influential to a CIED than out‐of‐field scattered photon doses. And, the CIED should be managed based on the expectation of the scattered neutrons, at these higher energies.

The data shown in Fig. 2 of the manuscript, to my opinion, are not conclusive to favor lead shield for higher primary energies (23 MV in this case), since the doses reported are very low and the use of lead does not improve things at distances beyond 5 cm (see Fig. 2 in the manuscript). The reduction seen at 2.5 cm distance in the Fig. 2 (from 7.5 cGy to 3.5 cGy for 100 MU), is only for the AP field and it may be due to scattered secondary electrons that reach 1.5 cm depth in tissue. These electrons can be mostly absorbed by placing a 5–10 mm tissue‐equivalent bolus on the CIED instead of a lead sheet. The out‐of‐field contamination at the surface is strongly dependent on the type of linac and linac head. At deeper depths and closer to the treatment field, out‐field doses are primarily from scatter within the treatment volume and depend on the treatment field size. The comparison between plan measured and calculated by the planning system out‐of‐field doses, as presented in Tables 1 and 3 and Figs. 2 and 3 of the manuscript, are referred to as large out‐of‐field distances, where most of the planning systems would severely underestimate the dose.^(7)^ Thus, again, no conclusions can be drawn on the benefit of the lead sheet over the CIED by the data presented.

In closing, the interpretation of the data by Bourgouin et al.^(1)^ is not well‐substantiated for the reasons presented in this letter, and the use of a thin sheet of lead on the CIED is not necessary since it will not prevent internal scatter dose to reach the device. However, it is best if a 5 mm tissue‐equivalent bolus is used to cover the CIED and *in vivo* dosimetry is implemented as part of the management policy for that category of patients.^(8)^ When higher than 10 MV photon beams are used, the presence of neutrons is a more serious issue that needs to be addressed for management of patients with a CIED. The authors did not discuss this important issue at all in their study.

## COPYRIGHT

This work is licensed under a Creative Commons Attribution 3.0 Unported License.
